# Point Defects Stability, Hydrogen Diffusion, Electronic Structure, and Mechanical Properties of Defected Equiatomic γ(U,Zr) from First-Principles

**DOI:** 10.3390/ma15217452

**Published:** 2022-10-24

**Authors:** Shasha Huang, Jiang-Jiang Ma, Kan Lai, Cheng-Bin Zhang, Wen Yin, Ruizhi Qiu, Ping Zhang, Bao-Tian Wang

**Affiliations:** 1 Institute of High Energy Physics, Chinese Academy of Sciences (CAS), Beijing 100049, China; 2 Department of Mechanical Engineering, City University of Hong Kong, Hong Kong, China; 3 Sino-French Institute of Nuclear Engineering and Technology, Sun Yat-sen University, Zhuhai 519082, China; 4 Spallation Neutron Source Science Center (SNSSC), Dongguan 523803, China; 5 Department of Basic Education, Tangshan University, Tangshan 063000, China; 6 Science and Technology on Surface Physics and Chemistry Laboratory, Mianyang 621908, China; 7 LCP, Institute of Applied Physics and Computational Mathematics, Beijing 100088, China; 8 Collaborative Innovation Center of Extreme Optics, Shanxi University, Taiyuan 030006, China

**Keywords:** uranium-zirconium alloy, defect, first-principles calculations, SQS

## Abstract

At present, many experimental fast reactors have adopted alloy nuclear fuels, for example, U-Zr alloy fuels. During the neutron irradiation process, vacancies and hydrogen (H) impurity atoms can both exist in U-Zr alloy fuels. Here, first-principles density functional theory (DFT) is employed to study the behaviors of vacancies and H atoms in disordered-γ(U,Zr) as well as their impacts on the electronic structure and mechanical properties. The formation energy of vacancies and hydrogen solution energy are calculated. The effect of vacancies on the migration barrier of hydrogen atoms is revealed. The effect of vacancies and hydrogen atom on densities of states and elastic constants are also presented. The results illustrate that U vacancy is easier to be formed than Zr vacancy. The H interstitial prefers the tetrahedral site. Besides, U vacancy shows H-trap ability and can raise the H migration barrier. Almost all the defects lead to decreases in electrical conductivity and bulk modulus. It is also found that the main effect of defects is on the U-5*f* orbitals. This work provides a theoretical understanding of the effect of defects on the electronic and mechanical properties of U-Zr alloys, which is an essential step toward tailoring their performance.

## 1. Introduction

Since the first exploration of the Clementine reactor in 1949, fast reactor development programs have been steadily advancing. They are considered to have potential contribution to sustainable development by saving uranium resources and reducing the long-term radiotoxicity of nuclear waste; and also considered as useful tools for the co-generation of hydrogen [1]. Metal alloy fuels were originally preferred to be employed in fast reactors due to their compatibility with sodium and their high fissile density [2]. Among all the metal alloy fuels, the uranium-plutonium-zirconium (U-Pu-Zr) ternary alloys are believed to be the most promising fuel because of their solidus temperature and compatibility with stainless steels [3,4,5,6]. The uranium-zirconium (U-Zr) binary alloys as the important subsystem of U-Pu-Zr are also of integral interest to fast reactor fuel development. Besides, the U-Zr alloy fuel has already been used in fast-spectrum reactors, such as the EBR-II. They are also continued to be attractive for advanced nuclear reactor concepts, such as the advanced sodium-cooled fast reactor [7,8]. The U-Zr alloy fuels are reported to possess high thermal conductivity, outstanding corrosion resistance, and the ability to contain high densities of fissile and fertile materials [2,7,8,9,10]. However, their safety and efficiency are nevertheless affected by issues like mechanical degradation and fuel swelling, which are closely related to irradiation damage.

Most of current research focus on the microstructural and thermodynamical properties of perfect U-Zr alloys. For example, the near-equilibrium microstructure and phase constituent of U-Zr alloys were investigated by Basak et al. [11] from experiments. The phase equilibria and decomposition temperature of the body-centered cubic (BCC) γ(U,Zr) were studied using the all-electron full-potential linear muffin-tin orbital method [12]. The thermal conductivity of γ(U,Zr) and other BCC binary uranium alloys was investigated via a combined ab-initio and empirical model [13]. A modified embedded-atom method potential was developed to study the structure, thermodynamics as well as the morphological evolution and segregation of U-Zr alloys [14,15]. Recently, the effect of temperature and Zr content on bulk modulus and heat capacity of γ(U,Zr) were calculated by Aly et al. [16] using ab initio molecular dynamics.

During the fission neutron irradiation, or even at the fabrication stage, some point defects, such as vacancies and hydrogen (H) atoms, are generated in the material [17,18,19,20,21]. These point defects can further form clusters and lead to adverse mechanical degradation [22,23,24] or even change the phase of nuclear fuel [25]. It is found that voids or dislocation loops formed in α-U can lead to the hardening of the material [26,27]. However, the effect of defects on the properties of U-Zr alloys has not been reported in the literature until recent years. The formation energy and migration barrier of vacancy and interstitial in α(U,Zr) were calculated by Huang et al. in 2011 [28]. Besides, they also investigated the defect clusters nucleation and diffusion behaviors in α(U,Zr) [29]. The threshold displacement energy of BCC γ-U and U-Zr alloy was determined by Beeler et al. [30] via interatomic potentials in 2018. Later, the effect of local atomic configuration on the vacancy formation energy in γ(U,Zr) was examined by Vizoso et al. using interatomic potential [31]. It was reported that the configuration of the nearest neighbor atoms has an essential influence on the value of vacancy formation energy in γ(U,Zr).

Apart from vacancy defects, H impurities can also lead to the degradation of materials, such as hydrogen embrittlement [32] and facilitated crack propagation by H diffusion [33]. Besides, the interaction between H atoms and uranium materials has long been a concern. Asada et al. [34] reported the H-absorption behavior in U-Zr alloys in 1995. They found that U-Zr alloys exhibit good H-absorption properties. However, the presence of H can drastically reduce the ductility and cause the plastic deformation of U and U-Zr alloys, even at low solubility [17,35]. Furthermore, it was also found that H solubility can be enhanced in Zr alloys under irradiation [36]. This might be deleterious to the material’s performance. Therefore, a sufficient knowledge of defect behavior on the atomic scale is necessary for the prediction of complex behavior of U-Zr alloys at a macroscopic level.

In 2013, Xie et al. [37] utilized the density functional theory (DFT) and DFT plus Hubbard parameter *U* (DFT + *U)* methods to analyze the structure, magnetic moments, and electronic structure of U metal and U-Zr alloys. The effects of spin-orbit coupling (SOC) were also considered in their work. They indicated that *U_eff_* = 1.24 eV should be used for both U and U-Zr alloys. However, different viewpoints have been proposed by Söderlind et al. [38,39]. They proved that neither the Hubbard *U* nor the SOC is necessary for correctly describing U metal and U-Zr alloys. Moreover, in our previous work [40], the influence of DFT and DFT + *U* methods on the physical properties of various U-Zr alloy phases was also compared. The result revealed that DFT + *U* will not bring significant difference to the structural, electronic, magnetic, and elastic properties of U-Zr alloys. Considering this fact, only the DFT method is adopted in the present work. In our previous work [40], we have investigated the physical properties of various U-Zr alloy phases, such as α-U and γ(U,Zr). As a promising candidate fuel for fast neutron reactors, U-Zr alloy will be exposed inevitably to the high-temperature environment. According to the most recent phase diagram provided by Okamoto [41], at the elevated working temperature of nuclear fuel, U-Zr alloy should be the BCC γ phase. The structural and mechanical behavior of ordered-γ(U,Zr) has been investigated in our previous work [40]. However, the structural disorder is very common in alloy systems [13,37]. The recent study also reveals that defect properties depend largely on the local atomic configuration [31]. Thus, this manuscript mainly focuses on the defect properties in disordered-γ(U,Zr). The results of ordered-γ(U,Zr) are also provided in Supplementary Materials. Please refer to Supplementary Materials for the detailed comparison of the ordered- and disordered-γ(U,Zr) defect properties.

To date, most of the studies on γ(U,Zr) focus on their morphology and thermodynamic properties. The defect behaviors in γ(U,Zr) were rarely reported in the literature. Moreover, the effects of defects on the electronic structure and mechanical properties also remain relatively unknown. Therefore, eight different defect types have been considered in this work through DFT approach. They are uranium vacancy ($V_{U}$), zirconium vacancy ($V_{Zr}$), two H substitutional systems: uranium lattice occupied by hydrogen atom ($H_{U}$) and zirconium lattice occupied by hydrogen atom ($H_{Zr}$), as well as four H interstitial systems: tetrahedral $H_{i}$ in perfect cell (perfect + T-site $H_{i}$), octahedral $H_{i}$ in perfect cell (perfect + O-site $H_{i}$), tetrahedral $H_{i}$ in $V_{U}$-containing cell ($V_{U}$ + T-site $H_{i}$), octahedral $H_{i}$ in $V_{U}$-containing cell ($V_{U}$ + O-site $H_{i}$), respectively. Their stability is examined by formation energy, and their diffusion behaviors are studied by calculating migration barrier. The effect of defects on electronic structure and mechanical properties of γ(U,Zr) are also calculated in this work. Our results reveal that the presence of $V_{U}$ can trap the diffusion of H atoms. Besides, the presence of defects leads to degradation of the electronical and mechanical performance of γ(U,Zr). This work provides a first theoretical understanding of the effect of defects on the electronic and mechanical properties of U-Zr alloys, which is an essential step toward tailoring their performance.

## 2. Computational Methods

### 2.1. Computational Details

The first-principles calculations based on the projected augmented wave (PAW) method [42,43] were performed using the Vienna ab initio simulation package (VASP) [44,45]. The Perdew-Burke-Ernzerhof (PBE) form of the generalized gradient approximation (GGA) [46] was used to describe the exchange-correlation interactions. We adopted a 3×3×3 BCC supercells (54 atoms) and a γ-centered 5×5×5 k-point mesh for both ordered-γ(U,Zr) and disordered-γ(U,Zr) in the calculations. The cutoff energy was set to 500 eV and all atoms were fully relaxed until the Hellmann-Feynman forces become less than 0.02 eV/Å. The uranium 6s^2^7s^2^6p^6^6d^2^5f^2^ and zirconium 4s^2^5s^1^4p^6^4d^3^ electrons were treated as valence electrons. The climbing image nudged elastic band (CI-NEB) method [47] was used to study the diffusion energy barrier of H interstitial in γ(U,Zr). Three intermediate images were inserted between the fixed endpoints with a spring constant of −5 eV/Å and the force convergent criteria was −0.03 eV/Å.

### 2.2. Simulation Models

The disordered supercell was generated by the special quasi-random structure (SQS) approach proposed by Zunger et al. [48]. This method helps to closely mimic the most relevant local pair as well as multisite correlation functions of the random substitutional alloys. Considering the fact that defect properties in the SQS structure might largely depend on the local environment of the defect [31], in this work, all the results for disordered-γ(U,Zr) were calculated from an average of 15 different configurations. More than ten SQS supercells have been tested, and the final adopted cells were selected based on energetic stability (i.e., the supercells which possess the lowest energies among all the models were selected). The detailed structures of the ordered-γ(U,Zr) supercell and the three SQS supercells are shown in Figure S1. For each SQS model, five different defect sites were selected to generate different local environment surrounding the defect. Thus, 15 different configurations for each defect type in disordered-γ(U,Zr) were studied in total.

## 3. Results and Discussion

**Figure 1:**
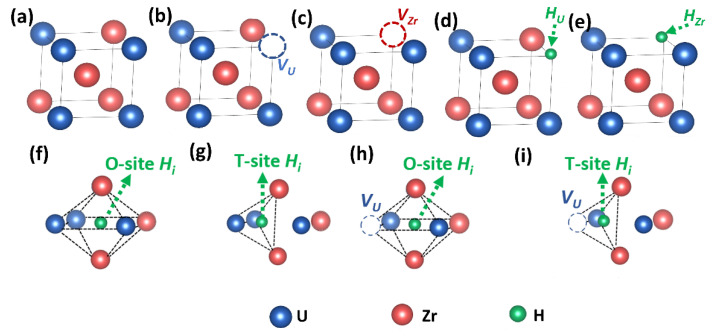
Schematic illustrations of (**a**) perfect unit cell, (**b**) $V_{U}$, (**c**) $V_{Zr}$, (**d**) $H_{U}$, (**e**) $H_{Zr}$, (**f**) perfect + O-site $H_{i}$ , (**g**) perfect + T-site $H_{i}$ (**h**) $V_{U}$ + O-site $H_{i},$ (**i**) $V_{U}$ + T-site $H_{i}$ models in γ(U,Zr).

During neutron irradiation process, vacancies and H impurity atoms can both be present in γ(U,Zr). Therefore, in this work, eight different defect types including uranium vacancy ($V_{U}$), zirconium vacancy ($V_{Zr}$), two H substitutional systems: uranium lattice occupied by hydrogen atom ($H_{U}$) and zirconium lattice occupied by hydrogen atom ($H_{Zr}$), as well as four H interstitial systems: tetrahedral $H_{i}$ in perfect cell (perfect + T-site $H_{i}$), octahedral $H_{i}$ in perfect cell (perfect + O-site $H_{i}$), tetrahedral $H_{i}$ in $V_{U}$-containing cell ($V_{U}$ + T-site $H_{i}$), octahedral $H_{i}$ in $V_{U}$-containing cell ($V_{U}$ + O-site $H_{i}$) are studied. We emphasize that, if without specific mention, all the following numerical results in this manuscript are the average value from 15 different configurations in three 3×3×3 SQS supercells (five configurations in each SQS supercell). However, in order to give readers a better understanding of the different defect structures studied, a schematic diagrams of the perfect unit cell as well as all the investigated defect systems in γ(U,Zr) in shown in Figure 1. The complete 3×3×3 SQS supercells employed in this work are shown in Figure S1.

### 3.1. Structure and Defect Stabilities

The structural stability of perfect γ(U,Zr) is studied via cohesive energy *E_cohesive_* [49], which can be defined as:

(1) $$E_{cohesive}=\frac{N_{U}\times E_{U}+N_{Zr}\times E_{Zr}-E\left[\gamma \left(U,\mathrm{Zr}\right)\right]}{N_{U}+N_{Zr}}$$

In the formula, $N_{U}\;$and $N_{Zr}$ are the numbers of U and Zr atoms in the supercells, respectively; $E_{U}$ and $E_{Zr}$ are the energies of an isolated U and Zr atom, respectively; *E[*γ(U,Zr)*]* is the total energy of the γ(U,Zr) supercells.

Using Equation (1), the calculated cohesive energy of the perfect ordered-γ(U,Zr) is 6.11 eV, while it is 6.49 eV for disordered-γ(U,Zr). These positive values clearly indicate that these two systems are both stable. The disordered-γ(U,Zr) is relatively more stable than the ordered one. Thus, in the following, we mainly focus on the defect properties in disordered-γ(U,Zr), and the corresponding result of ordered ones are provided in the Supplementary Materials.

After obtaining the structural stability of the perfect cell, $V_{U}$ or $V_{Zr}$ is added in the cell by deleting a corresponding atom. Their stability is studied via vacancy formation energy, which is explained in detail in Refs. [50,51]. In our γ(U,Zr) system, this formula can be expressed as:

(2) $$E_{f}\left(V_{X}\right)=E\left[\gamma \left(U,\mathrm{Zr}\right),V_{X}\right]-E\left[\gamma \left(U,\mathrm{Zr}\right)\right]+E\left(X\right)$$

where *E_f_(V_X_)* is the formation energy of *X* type vacancy (*X* can be U or Zr), *E[*γ(U,Zr)*,V_X_]* is the total energy of γ(U,Zr) supercell with a vacancy, and *E(X)* is the energy of an isolated X atom as defined in Ref. [51], X can be U or Zr.

By calculating the average formation energy value of 15 configurations for each type, we obtain 1.95 eV for $V_{U}$ and 2.20 eV for $V_{Zr}$ The lower formation energy of $V_{U}$ indicates that γ(U,Zr) is more inclined to produce $V_{U}$.

Since $V_{U}$ is easier to form than $V_{Zr}$, in the following study of interaction between vacancy and $H_{i}$, only the effect of $V_{U}$ is considered.

Before investigating the interaction between $V_{U}$ and $H_{i}$, it is necessary to firstly study the stability of H impurity atoms, which is also of great interest to many nuclear materials [36,52,53]. Their stability is assessed by hydrogen solution energy. The lower the solution energy is, the stabler the hydrogen defect type is. The solution energy of H atoms and H-vacancy complex defects were calculated respectively by the following equation as discussed in Ref. [54]:

(3) $$E_{s}=E\left[\gamma \left(U,Zr\right),H\right]-E\left[\gamma \left(U,Zr\right)\right]-\frac{1}{2}E\left(H_{2}\right)$$

and

(4) $$E_{s}=E\left[\gamma \left(U,Zr\right),V_{X},H\right]-E\left[\gamma \left(U,Zr\right),V_{X}\right]-\frac{1}{2}E\left(H_{2}\right)$$

where *E[*γ(U,Zr)*,H]* and *E[*γ(U,Zr)*,V_X_,H]* represent the energies of a γ(U,Zr) supercell with only H atom and with both H atom and vacancy, respectively. *E(H_2_)* denotes the energy of H_2_ molecular.

According to Equations (3) and (4), the solution energies of the two H substitutional systems as well as the solution energy of different $H_{i}$, in perfect or $V_{U}$-containing cell are calculated. The data are collected in Table 1. It can be seen that the solution energies of all the impurity positions are negative. Such results reveal that the hydrogen atoms can spontaneously occupy these positions in γ(U,Zr). The good solubility of H atoms in γ(U,Zr) was also reported in the experiment [34]. Besides, among all the data in Table 1, T-site $H_{i}$ always shows a lower solution energy than that of O-site $H_{i}$, indicating that the interstitial at T-site is more stable. Moreover, the result also depicts that $H_{Zr}$ system is more stable than $H_{U}$ system, and $H_{i}$ near a $V_{U}$ always possesses a lower solution energy than in a perfect cell. The results of vacancy formation energy and hydrogen solution energy in ordered-γ(U,Zr) are provided in Table S1.

Based on the above analysis of the defect stabilities, it can be seen that: $V_{U}$ is easier to be generated than $V_{Zr}$; T-site $H_{i}$ is more stable than O-site $H_{i}$; $H_{Zr}$ is more stable than $H_{U}$. Therefore, in the following analysis of the performance changes caused by defects, these relatively more stable defects were adopted for comparison.

**Figure 2:**
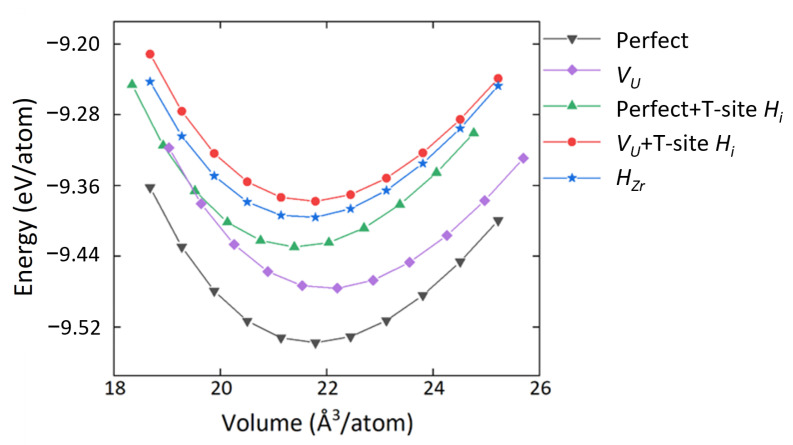
Energy (per atom) vs. volume (per atom) relationship for perfect and relatively more stable defect systems in γ(U,Zr).

The energy-versus-volume curves of these relatively more stable defect structures are shown in Figure 2 (the energy-volume curve of ordered-γ(U,Zr) is also displayed in Figure S2). As can be seen, all the defect structures would increase the energy of the system. And the equilibrium volume of the perfect cell in this work is 21.93 Å/atom, in good agreement with a previous γ(U,Zr) SQS model, where the value is 21.97 Å/atom [37].

### 3.2. Effect of Uranium Vacancy on H Diffusion

Many previous theoretical works have reported the H-trap behaviors of monovacancy [54,55]. Hence, in the present work, the H-trap behaviors of the mono-$V_{U}$ in γ(U,Zr) are also investigated. Besides, the effect of $V_{U}$ on the diffusion behavior of $H_{i}$ is illustrated utilizing the CI-NEB method [47].

In order to study the trapping behavior of single vacancy on hydrogen atom in ordered γ(U,Zr), the trapping energy of uranium vacancy on T-site $H_{i}$ is calculated. The trapping energy is defined as [54]:

(5) $$E_{H_{i}}^{trap}=E\left[\gamma \left(U,\mathrm{Zr}\right),V_{U},H\right]-E\left[\gamma \left(U,\mathrm{Zr}\right),V_{U}\right]-\left(E\left[\gamma \left(U,\mathrm{Zr}\right),H\right]-E\left[\gamma \left(U,Zr\right)\right]\right)$$

Among them, $E\left[\gamma \left(U,\mathrm{Zr}\right),V_{U},H\right]$ and $E\left[\gamma \left(U,\mathrm{Zr}\right),V_{U}\right]$ represent the total energy of a uranium vacancy-containing cell with and without a hydrogen interstitial, respectively; $E\left[\gamma \left(U,\mathrm{Zr}\right),H\right]$ and $E\left[\gamma \left(U,\mathrm{Zr}\right)\right]$ represent the total energy of a perfect cell with or without a tetrahedral hydrogen interstitial, respectively.

According to Equation (5), the trapping energy of T-site $H_{i}$ by $V_{U}$ is −0.24 eV. The negative values indicate that $H_{i}$ is more inclined to be trapped by $V_{U}$ than dispersed at different tetrahedral interstitial sites.

**Figure 3:**
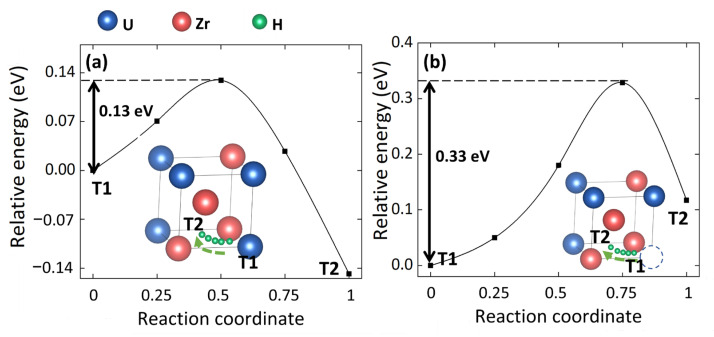
Representative energy barriers of T-site to T-site $H_{i}$ diffusion in (**a**) perfect and (**b**) $V_{U}$ -containing γ(U,Zr) cells. The green arrow in the figure shows the diffusion path of H atom. T1 and T2 represent the initial T-site and final T-site, respectively. The five green spheres indicate the positions of H atom in different images of CI-NEB.

Since $V_{U}$ can trap the T-site $H_{i}$, the effect of $V_{U}$ on the T-site to T-site diffusion of $H_{i}$ is further investigated. The average diffusion energy barriers of T-site $H_{i}$ in perfect cell and $V_{U}$-containing cell are 0.13 eV and 0.36 eV, respectively. Figure 3 shows two representative diffusion energy barriers, and the diffusion path for the T-site $H_{i}$ in perfect cell [Figure 3a] and $V_{U}$ containing cell [Figure 3b], respectively. The green arrow in the figure shows the diffusion path of T-site $H_{i}$. T1 and T2 represent the initial T-site and final T-site, respectively. The five green spheres indicate the positions of H atom in different images of CI-NEB.

Comparing Figure 3a,b, one can see that the presence of $V_{U}$ can remarkably lead to the increase in the diffusion energy barriers of T-site $H_{i}$. This phenomenon accords well with our previous conclusion that $V_{U}$ can trap the T-site $H_{i}$. Furthermore, it should also be mentioned that the average energy barrier increased by $V_{U}$ is about 0.23 eV, and the trapping energy in our work is −0.24 eV. This indicates that the diffusion energy barrier increased are mainly caused by the H-trap behaviors of $V_{U}$.

### 3.3. Electronic Structure

**Figure 4:**
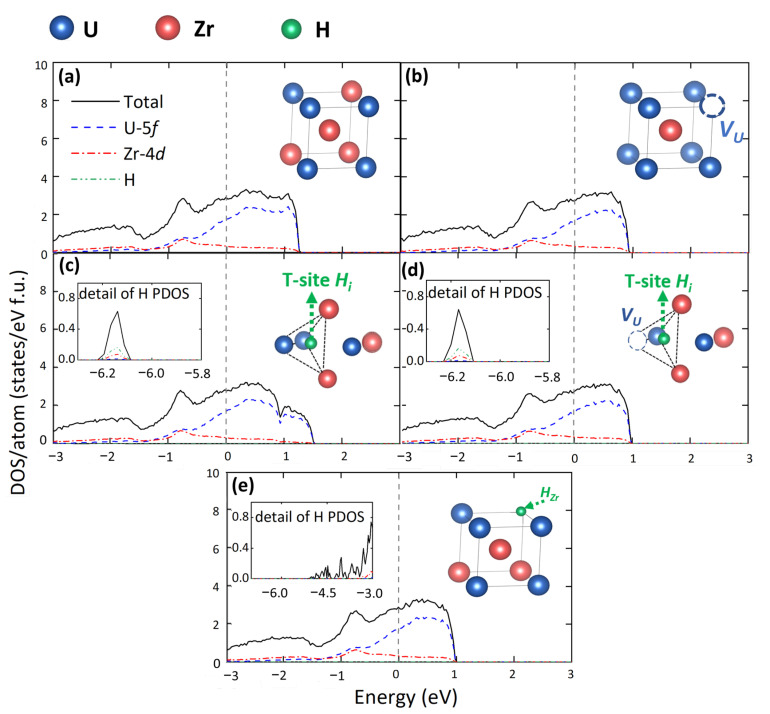
Comparison of the TDOS and PDOS of (**a**) perfect cell, (**b**)$\;V_{U}$, (**c**) perfect + T-site $H_{i}$, (**d**) $V_{U}$ + T-site $H_{i}$, and (**e**) $H_{Zr}$ models in γ(U,Zr). The Fermi level is set at zero.

It is well known that many macroscopical properties, such as elasticity, conductivity, and hardness, are closely related to the electronic structure. Previous work reported that vacancies can significantly affect the electronic structure of the materials, and further influence their performance [56]. Therefore, the description of electronic structure is necessary for the further study of the physical and chemical properties of γ(U,Zr). Here, the electronic total and partial densities of states (TDOS and PDOS) for different defected structures in γ(U,Zr) are illustrated in Figure 4. It should be mentioned that the DOS of 15 different configurations for each defect type were all studied but observe no great difference. Thus, in Figure 4, only the DOS of one of the 15 configurations for each defect type were presented. For all the structures, the TDOS near the Fermi energy level are mainly occupied by the U-5*f* orbitals. As can be seen in Figure 4, defects would not bring great difference to the DOS near the Fermi level. But the introduction of $H_{i}$ leads to a peak near −6.0 eV. Such kind of peak in deep energy level has also been observed in $H_{i}$ doped α-U [52]. However, this peak does not appear in the H substitutional systems.

In order to quantitatively compare the effect of defects on the TDOS at the Fermi level ($N\left(E_{F}\right)$) in γ(U,Zr), Table 2 listed the $N\left(E_{F}\right)$ of these defected structures. The table also shows the number of valence electrons of U and Zr elements at the Fermi energy level [$N_{e}\left(U_{f}\right)$ and $N_{e}\left(Zr_{d}\right)$] in different structures. All the values are the average value from 15 different configurations for each defect type. Meanwhile, the previous DFT-PBE results [13,37] related to the corresponding systems are also listed for comparison. One can see that our results are well consistent with those available data. Besides, except the two $H_{Zr}$ systems, all defects led to the reduce of $N\left(E_{F}\right)$. This means that those defects can induce the degradation of the electrical conductivity. The same trend is also found in $N_{e}\left(U_{f}\right)$. However, the $N_{e}\left(Zr_{d}\right)$ remains relatively unaffected by all the defects. Therefore, the main effect from defects is on U-5*f* orbitals. The comparison of the ordered and disordered structure on the electronic properties of γ(U,Zr) is provided in Figure S3 and Table S2 in the Supplementary Materials.

### 3.4. Mechanical Properties

The effect of defects on the mechanical properties is an important indicator in real applications of alloys fuels. In this work, the theoretical equilibrium volume and bulk modulus *B* were obtained by fitting the energy-volume data with the third-order Birch-Murnaghan equation of state (EOS) [57]. The elastic constants ($C_{11},C_{12},C_{44}$) were calculated by linear fitting of the stress-strain curves obtained from first-principles calculations. After obtaining the elastic constants, the bulk modulus *B* can also be calculated from the Voigt-Reuss-Hill (VRH) approximations [58,59,60]:$\;B=\frac{1}{2}\left(B_{R}+B_{V}\right)$. Detailed computation scheme of $B_{R}$ and $B_{v}$ was discussed in Ref. [61]. The data are collected in Table 3. To crosscheck the reliability of our results, the bulk modulus $B_{1}$ was obtained by using the VRH approximation, as well as the corresponding elastic constants are also listed. For comparison, the available data from other experimental works or DFT calculations [13,37,62] are also presented in Table 3. From Table 3, it can be seen that the calculated $V_{0}$ of perfect cells are comparable with available experiment [62] as well as previous DFT calculations [13,37]. Besides, the bulk modulus obtained by two different methods (EOS fitting and VRH approximation) in this work are also close to each other, which means that our calculations are self-consistent. Comparing the effect of defects on the three elastic constants, one can find that the $C_{11}$ is most affected by defects. Except for the perfect + T-site $H_{i}$ structure, other defects all lead to the decrease in bulk moduli.

For cubic crystals, the mechanical stability criteria are:$C_{11}>0,C_{44}>0,C_{11}>\left|C_{12}\right|,\left(C_{11}+2C_{12}\right)>0$. As shown in Table 3, all our investigated structures possess a larger $C_{12}$ than $C_{11}$, which violating the criterion of ($C_{11}$>|$C_{12}|$). Thus, they are all mechanically unstable. Such instability of the γ(U,Zr) was also found in other DFT works on BCC γ-U [63,64,65] and BCC β-Zr [66,67]. After all, the BCC structure is the high-temperature phase for U-Zr systems. The low-temperature instability can be expected. We present such results because the defects effects on the mechanical properties can be derived from them. The effect of defects on the mechanical properties in ordered-γ(U,Zr) is illustrated in Table S3.

## 4. Conclusions

In summary, through first-principles calculations, the defect behaviors and the effect of defects on the electronic structure and mechanical properties of γ(U,Zr) are investigated. Our calculated structural, electronic structure, and mechanical properties of the perfect γ(U,Zr) are consistent with previous DFT and experimental works. The results show that, in γ(U,Zr), uranium vacancies possess lower formation energy than zirconium vacancies (1.95 eV vs. 2.20 eV). The tetrahedral hydrogen interstitials have the lowest solution energy than any other types of hydrogen impurity defects. Besides, with the presence of uranium vacancy, the migration barrier of hydrogen atoms can be increased by 0.23 eV in accordance with the trapping energy of uranium vacancy on T-site $H_{i}$ (−0.24 eV). In addition, in analyzing of the electronic structure, it is found that the main electronic occupation near the Fermi energy level is from the U-5*f* orbitals. Finally, the effect of defects on mechanical properties of γ(U,Zr) is investigated. The results reveal that almost all the defects can cause mechanical degradation. Based upon the present results, we will study the effect of temperature on the stability, thermodynamic and mechanical properties of the U-Zr alloys in our next work. This work provides a theoretical understanding of the effect of defects on the electronic and mechanical properties of U-Zr alloys, which is an essential step toward tailoring their performance. Besides, the defect formation energy and migration energy calculated in the present work could be a reference data for the improvement of interatomic potential for U-Zr alloys.

## Figures and Tables

**Table 1:** Solution energies of different hydrogen doping sites in γ(U,Zr), including H substitutional systems and interstitial sites with or without $V_{U}$.

	Disordered *E_s_* (eV)
$H_{U}$	−0.52
$H_{Zr}$	−0.67
perfect + T-site $H_{i}$	−0.89
perfect + O-site $H_{i}$	−0.64
$V_{U}$ + T-site $H_{i}$	−1.13
$V_{U}$ + O-site $H_{i}$	−0.76

**Table 2:** Comparison of the TDOS at the Fermi level $N\left(E_{F}\right)$, and the number of uranium and zirconium valence electrons at the Fermi level [$N_{e}\left(U_{f}\right)$ and $N_{e}\left(Zr_{d}\right)$] in different defect systems. For comparison, data from other DFT-PBE works [13,37] are also listed. All the units are in states/eV/atom.

	$N\left(E_{F}\right)$	$N_{e}\left(U_{f}\right)$	$N_{e}\left(Zr_{d}\right)$
$\mathrm{Perfect}\;\left(\mathrm{Ours}\right)$	2.83	1.72	0.35
$\mathrm{Perfect}\;\left(\mathrm{Refs}.\right)$	2.52 [37]	1.58 [37], 1.63 [13]	0.37 [37]
$V_{U}$	2.86	1.70	0.35
perfect + T-site $H_{i}$	2.82	1.68	0.33
$V_{U}$ + T-site $H_{i}$	2.81	1.64	0.34
$H_{Zr}$	2.90	1.71	0.32

**Table 3:** Calculated equilibrium volume $V_{0}$, elastic constants $C_{11},\;C_{12},\;and\;C_{44}$, and bulk modulus $B_{1}$ obtained by VRH approximation, $B_{2}$ obtained by fitting the third-ordered Birch-Murnaghan EOS.

	$V_{0}\;\left({Å}^{3}/atom\right)\;$	$C_{11}\;\left(GPa\right)\;$	$C_{12}\;\left(GPa\right)$	$C_{44}\;\left(GPa\right)$	$B_{1}\;\left(GPa\right)$	$B_{2}\;\left(GPa\right)$
$\mathrm{Perfect}\;\left(\mathrm{Ours}\right)$	21.93	75.2	120.8	36.7	105.6	103.2
$\mathrm{Perfect}\;\left(\mathrm{Refs}.\right)$	21.97 [37], 21.81 [13], 22.29 [62]	-	-	-	-	-
$V_{U}$	22.22	67.3	114.9	34.8	99.0	99.5
perfect + T-site $H_{i}$	21.65	73.8	122.6	38.7	106.3	103.6
$V_{U}$ $+T-\mathrm{site}\;H_{i}$	21.89	69.3	116.9	34.7	101.0	100.2
$H_{Zr}$	21.78	73.3	112.6	38.7	99.5	100.8

## Data Availability

The data presented in this study are available on request from the corresponding authors. The data are not publicly available due to ongoing research in the project.

## Supplementary Materials

The following supporting information can be downloaded at: https://www.mdpi.com/article/10.3390/ma15217452/s1, Figure S1: Schematic illustration of (a) supercell of ordered-γ(U,Zr), and (b–d) are the three different SQS models employed in this work; Table S1: Formation energy of *V_U_* and *V_Zr_*, and the solution energy of H atom in ordered-γ(U,Zr); Figure S2: Energy-versus-volume curves for different defect systems in ordered-γ(U,Zr); Figure S3: Comparison of TDOS and PDOS of different defected structures in ordered-γ(U,Zr). Table S2: The *N(E_F_)*, and the number of uranium and zirconium valence electrons at the Fermi level *N_e_(U_f_)* and *N_e_(Zr_d_)* in ordered-γ(U,Zr); Table S3: Calculated equilibrium volume *V_0_*, elastic constants, and bulk modulus *B*_1_ obtained by VRH approximation, *B*_2_ obtained by fitting the third-ordered Birch-Murnaghan EOS. of different defected ordered-γ(U,Zr). References [37,40,66,68,69,70,71,72,73] are cited in the Supplementary Materials.

## Abbreviations

The parameters and abbreviations used in the present work are summarized in this section according to the order in which they appear in the text.

U-Pu-Zr	Uranium-plutonium-zirconium
U-Zr	Uranium-zirconium
BCC	body-centered cubic
H	Hydrogen
DFT	Density functional theory
DFT + U	Density functional theory plus Hubbard parameter U
SOC	Spin-orbit coupling
$V_{U}$	Uranium vacancy
$V_{Zr}$	Zirconium vacancy
$H_{U}$	Uranium lattice occupied by hydrogen atom
$H_{Zr}$	Zirconium lattice occupied by hydrogen atom
$\mathrm{Perfect}+O-\mathrm{site}\;H_{i}$	Octahedral hydrogen interstitial in perfect cell
$\mathrm{Perfect}+T-\mathrm{site}\;H_{i}$	Tetrahedral hydrogen interstitial in perfect cell
$V_{U}$$+O-\mathrm{site}\;H_{i}$	Octahedral hydrogen interstitial in uranium vacancy-containing cell
$V_{U}$$+T-\mathrm{site}\;H_{i}$	Tetrahedral hydrogen interstitial in uranium vacancy-containing cell
CI-NEB	Climbing image nudged elastic band
SQS	Special quasi-random structure
*E_cohesive_*	Cohesive energy
$N_{U}$	Number of uranium atoms
$N_{Zr}$	Number of zirconium atoms
$E_{U}$	Energy of an isolated uranium atom
$E_{Zr}$	Energy of an isolated zirconium atom
*E[γ(U,Zr)]*	Total energy of the γ(U,Zr) supercells
$E_{f}\left(V_{X}\right)$	Formation energy of X type vacancy, X can be uranium or zirconium
$E\left[\gamma \left(U,Zr\right),V_{X}\right]$	Total energy of γ(U,Zr) supercells with a vacancy
$E\left(X\right)$	Energy of an isolated X atom, X can be uranium or zirconium
$E_{S}$	Hydrogen solution energy
*E[γ(U,Zr),H]*	Total energy of the γ(U,Zr) supercells with H atom
$E\left(H_{2}\right)$	Energy of H2 molecular
$E\left[\gamma \left(U,Zr\right),V_{X},H\right]$	Total energy of the γ(U,Zr) supercells with both X type vacancy and H atom, X can be uranium or zirconium
$E_{H_{i}}^{trap}$	Trapping energy of uranium vacancy on T-site $H_{i}$
TDOS	Total densities of states
PDOS	Partial densities of states
$N\left(E_{F}\right)$	TDOS at the Fermi level
$N_{e}\left(U_{f}\right)$	Number of valence electrons of U element at the Fermi energy level
$N_{e}\left(Zr_{d}\right)$	Number of valence electrons of Zr element at the Fermi energy level
EOS	Equation of state
VRH approximations	Voight-Reuss-Hill approximations
