# Supplementary material for: Point Defects Stability, Hydrogen Diffusion, Electronic Structure, and Mechanical Properties of Defected Equiatomic γ(U,Zr) from First-Principles
Source: Materials (Basel). 2022 Oct 24;15(21):7452. doi: 10.3390/ma15217452 (PMC9656540; doi:10.3390/ma15217452)
Supplement: Supplementary file 1 [file materials-15-07452-s001.zip › Supplementary_material_revised.pdf]

Supplementary material

Point Defects Stability, Hydrogen Diffusion, Electronic Structure, and Mechanical Properties of Defected Equiatomic γ(U,Zr) from First-Principles

Shasha Huang [1,2,3], Jiang-Jiang Ma [1,4], Kan Lai [3], Cheng-Bin Zhang [5], Wen Yin [1,4], Ruizhi Qiu [6], Ping Zhang [7,*], and Bao-Tian Wang [1,4,8,*]

1 Institute of High Energy Physics, Chinese Academy of Sciences (CAS), Beijing 100049, China
2 Department of Mechanical Engineering, City University of Hong Kong, Hong Kong, China
3 Sino-French Institute of Nuclear Engineering and Technology, Sun Yat-sen University, Zhuhai 519082, China
4 Spallation Neutron Source Science Center (SNSSC), Dongguan 523803, China
5 Department of Basic Education, Tangshan University, Tangshan 063000, China
6 Science and Technology on Surface Physics and Chemistry Laboratory, Mianyang 621908, China
7 LCP, Institute of Applied Physics and Computational Mathematics, Beijing 100088, China
8 Collaborative Innovation Center of Extreme Optics, Shanxi University, Taiyuan 030006, China
* Correspondence: zhang_ping@iapcm.ac.cn (P. Z.); wangbt@ihep.ac.cn (B-T. W.)

Figure S1 shows the supercell of ordered-γ(U,Zr) and the three special quasi-random structure (SQS) simulation cells employed in our work.

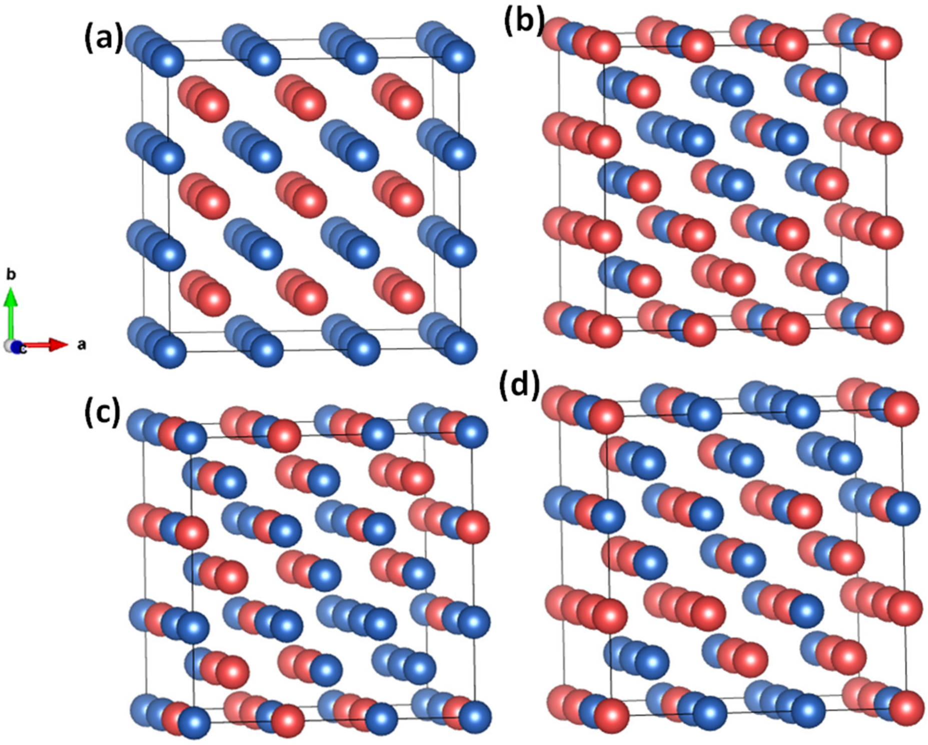

Figure S1. Schematic illustration of (a) supercell of ordered-γ(U,Zr), and (b)-(d) are the three different SQS models employed in this work.

The formation energy of V_U and V_{Zr}, and the solution energy of H atom in ordered-γ(U,Zr) are collected in Table S1. By comparing with the formation energy of vacancies in disordered-γ(U,Zr), one can find that, the formation energies for both V_U and V_{Zr} in disordered-γ(U,Zr) are always higher

than those in ordered-γ(U,Zr). Thus, it is more difficult to produce vacancy defects in disordered-γ(U,Zr). Besides, our H solution results also depict that the H impurity atoms are less likely to stay in disordered-γ(U,Zr). Furthermore, the relative stability of these defects shows the same tendency as in disordered-γ(U,Zr), that is, V_U is easier to be generated than V_{Zr}; T-site H_i is more stable than O-site H_i; H_{Zr} is more stable than H_U. Therefore, in the following, we also only consider the effect of these relatively more stable defects on the properties of ordered-γ(U,Zr).

Table S1. Formation energy of V_U and V_{Zr} and solution energy of H atom in ordered-γ(U,Zr)

	**Formation energy E_f (eV)**	**Solution energy E_s (eV)**
V_U	0.66	-
V_{Zr}	1.80	-
H_U	-	−0.39
H_{Zr}	-	−0.99
Perfect+T-site H_i	-	−1.23
Perfect+O-site H_i	-	−0.89
V_U +T-site H_i	-	−1.25
V_U+O-site H_i	-	−0.91

In Figure S2, we give the energy-versus-volume curves of the relatively more stable defects in ordered-γ(U,Zr). Meanwhile, we also plot the data of the perfect ordered-γ(U,Zr) in our previous work [1] for comparison. Our present data agree well with our previous work. By comparing the results of disordered-γ(U,Zr), it can be seen that the energies of the five structures of the disordered system are all lower than those of the ordered system. The equilibrium volumes of the disordered system are also smaller those corresponding ordered system.

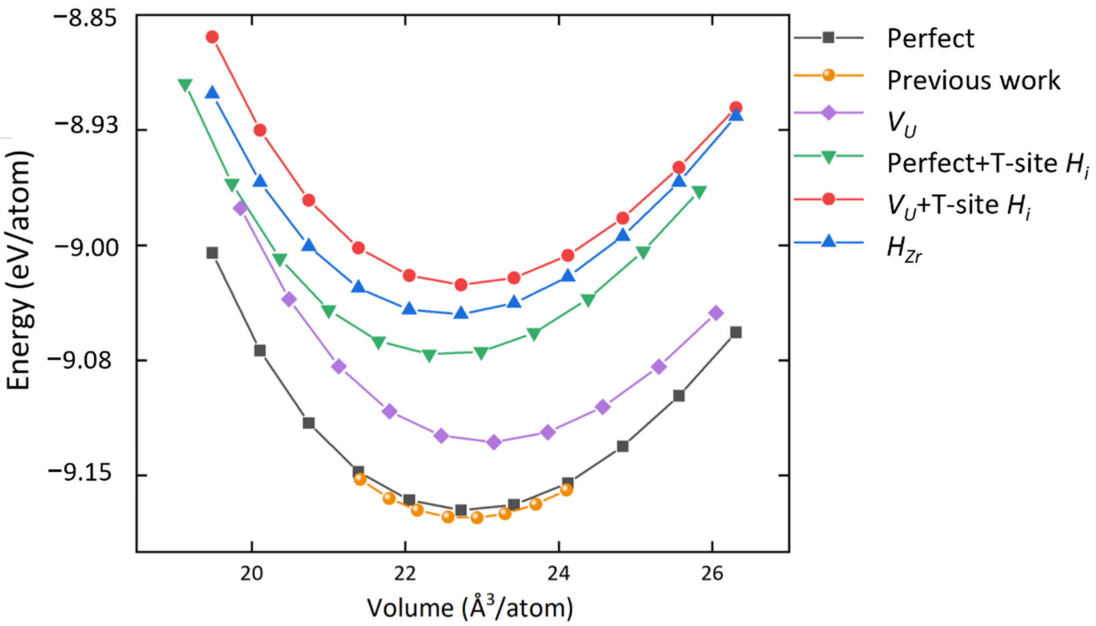

Figure S2. Energy-versus-volume relationship for different defect systems in ordered-γ(U,Zr).

Comparing the PDOS of ordered- and disordered-γ(U,Zr), one can clearly see the following features: (i) in ordered-γ(U,Zr), the Zr-4*d* orbitals present a peak near the Fermi energy level (this peak is also found to be near the Fermi energy level in previous work of perfect ordered-γ(U,Zr) [1]). However, such peak is close to −1 eV in disordered-γ(U,Zr) (also found in previous perfect disordered-γ(U,Zr) [2]); (ii) the electronic density of states at the Fermi level $N(E_F)$ in disordered-γ(U,Zr) is lower than that in ordered-γ(U,Zr). This reveals that the disordered-γ(U,Zr) is more stable than the ordered one, according well with our cohesive energy results. Besides, the $N(E_F)$ in previous perfect SQS model by Xie *et al.* [2] is also lower than that in perfect ordered-γ(U,Zr) in our previous work [1]. Moreover, in our present study, the full width at half maxima (FWHM) at Fermi level in disordered-γ(U,Zr) is about 2.04 eV, while it is only about 0.56 eV for ordered one. In the study of Xie *et al.* [2], the FWHM of the perfect disordered-γ(U,Zr) is about 1.76 eV while in the work of perfect ordered-γ(U,Zr) [1], the FWHM is about 0.48 eV. Therefore, the FWHM of the disordered-γ(U,Zr) is always larger than that of the ordered-γ(U,Zr). This means that the electrons near the Fermi level in the disordered-γ(U,Zr) is not that localized as the ordered one. Here, we believe that the disordered-γ(U,Zr) can describe the itinerant feature of the U-5*f* electrons more reasonably than the ordered one. After all, unlike the localized characters of the U-5*f* electrons in UO_2 [3], the itinerant features are always exhibited in U metal [4-6] and U-based alloys [7-9].

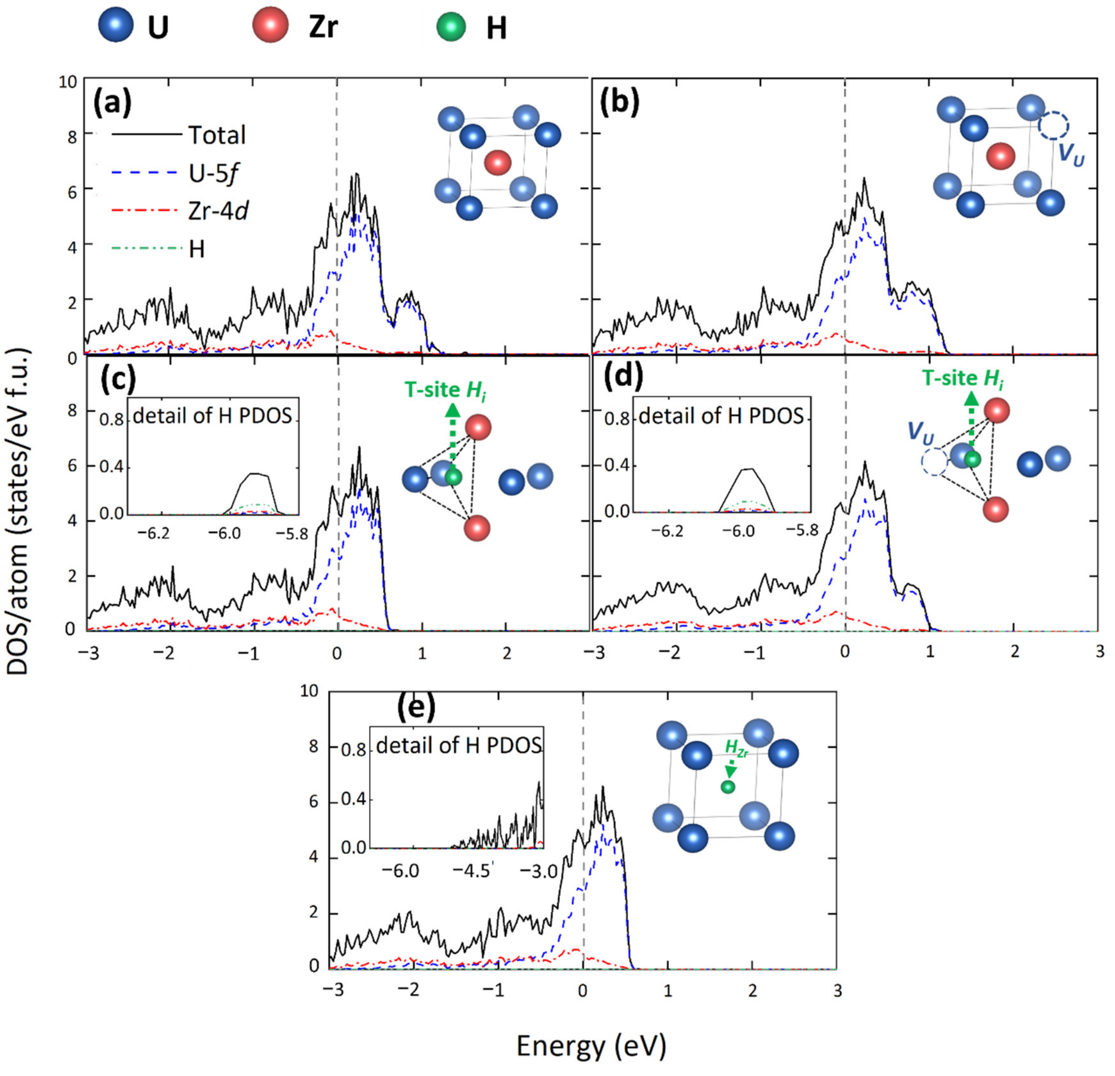

Figure S3. Comparison of the TDOS and PDOS of (**a**) perfect cell, (**b**) V_U, (**c**) perfect + T-site H_i, (**d**) V_U + T-site H_i, and (**e**) H_{Zr} model in ordered-γ(U,Zr). The Fermi level is set at zero.

Table. S2 shows the effect of defects on $N(E_F)$, and the number of uranium and zirconium valence electrons at the Fermi level $N_e(U_f)$ and $N_e(Zr_d)$ in ordered system. It can be found that the effect of defects in ordered-γ(U,Zr) is similar to the disordered system.

Table S2. Comparison of the $N(E_F)$, and the number of uranium and zirconium valence electrons at the Fermi level $N_e(U_f)$ and $N_e(Zr_d)$ in ordered system. All the units are in *states/eV/atom*. For comparison, data from our previous DFT work [1] are also listed.

	$N(E_F)$	$N_e(U_f)$	$N_e(Zr_d)$
Perfect (Ours)	4.76	3.00	0.58
Perfect (Ref.)	4.32 [1]	2.91 [1]	0.56 [1]
V_U	4.57	2.90	0.55
Perfect + T-site H_i	4.68	2.89	0.55
V_U + T-site H_i	4.73	2.96	0.55
H_{Zr}	4.82	3.01	0.54

Table. S3 presents the equilibrium volume V_0, elastic constants, and bulk modulus of different defected ordered-γ(U,Zr) systems. It can be found that the bulk moduli of the disordered-γ(U,Zr) are all stronger than those of the ordered-γ(U,Zr), according well with the smaller volumes found in disordered-γ(U,Zr).

Table S3. Calculated equilibrium volume V_0, elastic constants, and bulk modulus B_1 obtained by VRH approximation, B_2 obtained by fitting the third-ordered Birch-Murnaghan EOS of different defected ordered-γ (U,Zr). For comparison, data from our previous DFT work [1] are also listed.

	V_0 ($Å^3$/atom)	C_{11} (GPa)	C_{12} (GPa)	C_{44} (GPa)	B_1 (GPa)	B_2 (GPa)
Perfect (Ours)	22.86	60.1	106.0	15.9	90.7	86.8
Perfect (Ref.)	22.81[1]	55.2[1]	106.8[1]	7.8[1]	89.6[1]	88.9[1]
V_U	23.07	44.7	105.8	22.7	85.5	85.5
Perfect + T-site H_i	22.50	62.7	104.8	17.9	90.8	86.9
V_U + T-site H_i	22.76	45.1	105.4	23.2	85.3	85.2
H_{Zr}	22.57	46.0	104.0	17.4	84.7	86.2

References

1. Zhang, C.-B., et al., *Structural, electronic, and elastic properties of equiatomic UZr alloys from first-principles.* Journal of Nuclear Materials, 2017. **496**: p. 333-342.
2. Xie, W., et al., *Correlation and relativistic effects in U metal and U-Zr alloy: Validation ofab initioapproaches.* Physical Review B, 2013. **88**(23).
3. Wang, B.-T., et al., *First-principles calculations of phase transition, elastic modulus, and*

superconductivity under pressure for zirconium. Journal of Applied Physics, 2011. **109**(6).

4. Moore, K.T. and G. van der Laan, *Nature of the 5f states in actinide metals.* Reviews of Modern Physics, 2009. **81**(1): p. 235-298.
5. Adak, S., et al., *Uranium at high pressure from first principles.* Physica B: Condensed Matter, 2011. **406**(17): p. 3342-3347.
6. Tateiwa, N., et al., *Itinerant ferromagnetism in actinide $5f$-electron systems: Phenomenological analysis with spin fluctuation theory.* Physical Review B, 2017. **96**(3): p. 035125.
7. Kaneko, K., et al., *Large orbital magnetic moment and its quenching in the itinerant uranium intermetallic compounds $UTGa_5$ (T=Ni,Pd,Pt).* Physical Review B, 2003. **68**(21): p. 214419.
8. Fujimori, S.-i., et al., *Itinerant magnetism in URhGe revealed by angle-resolved photoelectron spectroscopy.* Physical Review B, 2014. **89**(10): p. 104518.
9. Schneider, W.-D. and C. Laubschat, *Actinide---noble-metal systems: An x-ray-photoelectron-spectroscopy study of thorium-platinum, uranium-platinum, and uranium-gold intermetallics.* Physical Review B, 1981. **23**(3): p. 997-1005.
